# Household food diversity and nutritional status among adults in Brazil

**DOI:** 10.1186/1479-5868-8-22

**Published:** 2011-03-27

**Authors:** Ilana N Bezerra, Rosely Sichieri

**Affiliations:** 1 Department of Epidemiology, Institute of Social Medicine, State University of Rio de Janeiro, Rua São Francisco Xavier, 524, 7° andar, Bloco E. Cep 20550-012, Rio de Janeiro, RJ, Brazil

## Abstract

**Background:**

The aims of this study were to evaluate whether a diversity of healthy foods in a household would decrease the availability of unhealthy foods and to evaluate the association between a healthy dietary diversity score (DDS) and nutritional status among adults.

**Methods:**

Data from the 2002-2003 Brazilian Household Budget Survey were used. This nationwide survey used a two-stage sampling technique: households were selected after selection of primary sample units (PSUs). Analyses were based on 3,393 PSUs, evaluating 659,816 records of food items purchased by 35,237 households. The DDS was based on the healthy food groups according to Brazilian food guidelines. Per capita acquisition of sugar, sweets, sugar-sweetened beverages and crackers, cookies and cakes (unhealthy food groups) in PSUs was also calculated. Individual weight and height were measured at household. Multivariate linear regression models estimated the association of underweight and overweight and obesity (excess weight) with the PSUs' DDS.

**Results:**

Greater acquisition of unhealthy food groups was associated with higher DDS. A high PSU's DDS was negatively associated with underweight (β = -0.38; p-value = 0.04) and positively associated with excess weight (β = 0.98; p-value = 0.05) after adjustment for availability of unhealthy food groups and socioeconomic variables.

**Conclusions:**

Our data indicate that there was no replacement of unhealthy food groups by healthy food groups, therefore a healthy diet message for obesity prevention should be combined with a message focused on eating less.

## Background

Obesity is a worldwide phenomenon that has reached both developed and developing countries, contributing to the development of chronic diseases as diabetes, cardiovascular diseases, and cancer [1]. The national prevalence of obesity (Body Mass Index (BMI) ≥ 30 kg/m^2^) in Brazilian adults (≥20 years) reached 15% in the most recent survey [2]. From 1975 to 2009, the prevalence of obesity increased four-fold among men (from 3% to 12%) and two-fold among women (from 8% to 17%) [2].

Trends in food availability in Brazilian households in the last three decades reveal that diverse traditional foods have been replaced by industrialized convenience foods [3]. The monotony of traditional diets could be one of the factors associated with their role in preventing weight gain [4,5]. In poor areas of developing countries, diets are based on few staple foods [6]. However, dietary diversity has been used to reflect the quality of the diet [7], and it has been associated with better health outcomes, especially with regard to issues of underweight among children [8,9]. Also, some studies have found a high correlation between a diverse diet and nutrient adequacy among adults and adolescents [10-13]. Therefore, food guidelines have emphasized the value of a diverse dietary pattern as a way to reach a healthy diet. Many countries, including Brazil, include a recommendation of a varied diet in their national dietary guidelines [14]. However, a diverse diet has been shown to be directly associated with greater energy intake [15]. Hence, the association between dietary diversity and obesity remains unclear.

Dietary diversity score (DDS) is usually calculated based on the number of different food groups consumed over a given period and has been used as a good indicator of diet quality; however, there is no established recommendation regarding the number of food groups considered in the DDS and how to deal with the amount of intake [16]. Another important issue is the inclusion of unhealthy food groups in the DDS [10].

Although some studies investigate the effect of a diverse diet on nutrient quality [17-19], there is little emphasis on the impact of the intake of a variety of healthy food items on the intake of unhealthy food items. Would greater availability of fruits and vegetables, for example, reduce the availability of unhealthy items?

We analyzed data from the Brazilian Household Budget Survey (HBS) to evaluate the following: 1) whether a diverse availability of healthy food items would decrease the availability of unhealthy food groups and 2) whether there is an association between DDS and nutritional status.

## Methods

### Population

We evaluated data from the 2002-2003 Brazilian HBS, which was carried out by the Brazilian Census Bureau on a national sample of about 50,000 households. A two-stage sampling technique was applied. In the first stage, the primary sampling units (PSU) were selected by systematic sampling with a probability proportional to the number of households in each PSU. In the second stage, the households were selected by simple random sampling. The survey was designed to include a representative sample of all five Brazilian regions, urban and rural areas, and socioeconomic levels. For this analysis, we included only urban areas.

The survey collected detailed information regarding all family expenditures on food purchased for home consumption during a one-week period. It covered a one-year period to ensure that all seasonal variation was captured. Since a seven-day period is not sufficient to evaluate household food availability, we consider PSUs as the unit of analysis. The mean number of households in the PSUs was 10, and they were homogenous regarding socioeconomic status.

**Figure 1:**
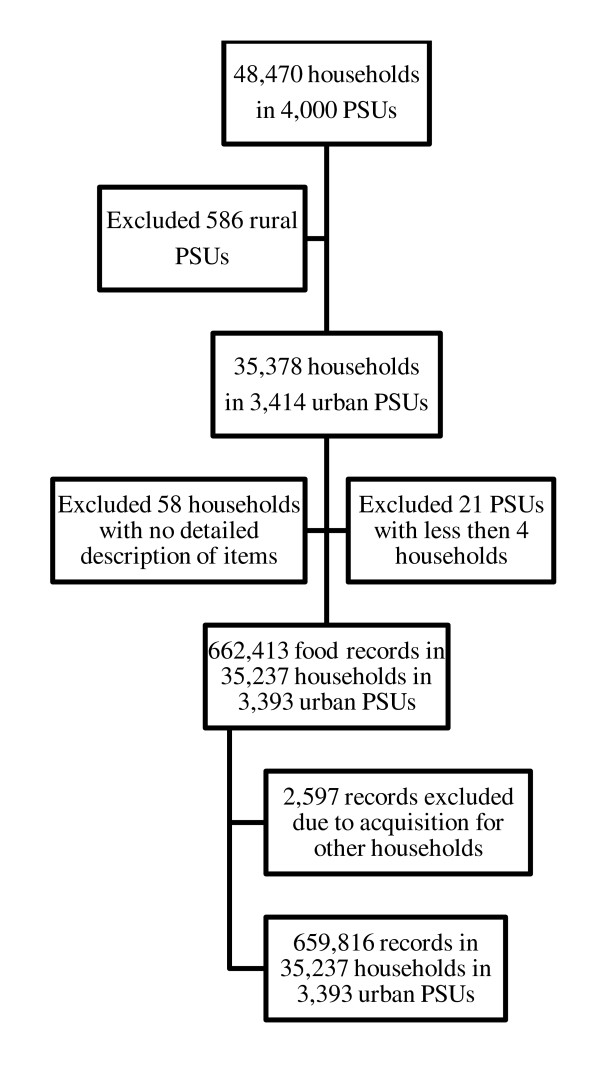
Sample definition

The records included a detailed description of the types of food acquired as well as the amount, the cost and the place of purchase. We excluded households for which a detailed description of all foods acquired was not available (n = 58). Also, PSUs with less than four households were excluded (n = 21). Items purchased by a household, but were to be consumed at another destination, were not considered (2,597 records). Thus, we analyzed the records of 659,816 food items purchased by 35,237 households situated in 3,393 urban PSUs. (See Figure 1 for sample definition.)

## Measurements

### 1. Food groups

All food information was collected using an open list of foods, allowing for the description of all items reported. Reported foods were categorized into the following 27 food items or food groups: rice, bread, other cereal, pulses, potatoes, carrots and pumpkin, manioc and other roots and tubers, coconuts, nuts, tomatoes, lettuce, other vegetables, bananas, oranges, other fruits, meats, seafood, poultry, eggs, milk, cheeses, other dairy products, oils and fats, sugar, sweets, sugar-sweetened beverages, and crackers, cookies and cakes. These groups were chosen based on Brazilian Food Guidelines, which recommend consuming a variety of items from the first twenty-three groups and avoiding the consumption of sugar, sweets, sugar-sweetened beverages, and crackers, cookies and cakes.

Acquisition of each food group by PSUs was transformed into dummy variables (1/0) to indicate whether the PSU acquired items from a particular group. Then, we calculated the number and the percentage of PSUs that acquired each food group (irrespective of the amount of food acquired) (Table 1). We also calculated the daily per capita acquisition (in grams) of sugar, sweets, sugar-sweetened beverages, and crackers, cookies and cakes in PSUs, dividing the amount acquired of these items in PSUs by the number of individuals in each PSU.

**Table 1:** Number, frequency (%) and 95% confidence interval (95% CI) of Primary Sample Units (PSU) that purchased food items and food groups

Food Group	Number N = 3393	Weighted frequency (95% CI)
Rice	3276	94.8 (93.1-96.4)
Bread	3383	99.9 (99.8-100)
Other cereals	3342	98.4 (97.6-99.2)
Pulses	3226	94.1 (92.4-95.8)
Potatoes	2976	92.2 (91.0-93.4)
Carrot and pumpkin	2589	79.9 (77.5-82.3)
Manioc and other roots and tubers	2116	59.6 (56.6-62.6)
Coconuts	978	27.1 (24.4-29.7)
Nuts	329	12.2 (10.2-14.3)
Tomato	3159	93.5 (92.0-94.9)
Lettuce	2353	77.7 (75.5-79.9)
Other vegetables	3297	97.9 (97.3-98.6)
Banana	3070	91.2 (89.4-92.9)
Orange	2462	76.6 (74.2-79.1)
Other fruits	3107	92.7 (91.1-94.3)
Meat	3370	99.1 (98.5-99.7)
Seafood	2099	58.1 (55.1-61.1)
Poultry	3298	97.7 (96.9-98.6)
Egg	3393	100 (100-100)
Milk	3386	99.7 (99.3-100)
Cheese	2531	83.8 (81.9-85.7)
Other dairy products	3276	97.8 (97.1-98.5)
Oils and fats	3350	98.6 (97.8-99.3)

Brazil - urban area, 2002-2003.

### 2. Dietary diversity score (DDS)

Dietary diversity score refers to the number of food groups purchased by the PSU (irrespective of the number of food items purchased in each group). For this score, we considered only the first twenty-three groups (i.e., healthy food groups). Therefore, this score can vary from 0 to 23, depending on the number of groups purchased in the PSU.

### 3. Anthropometric measurements

Weight was measured in the households to the nearest 100 g on electronic portable scales with a weight capacity of 150 kg. Height was measured to the nearest 5 mm with a vertical wall-mounted stadiometer. Individuals from 20 to 65 years old were classified as underweight (BMI < 18.5 kg/m^2^), normal weight (18.5 kg/m^2 ^≤ BMI < 25 kg/m^2^), or excess weight (BMI ≥ 25 kg/m^2^). Individuals with a BMI less than 15 kg/m^2 ^or more then 50 kg/m^2 ^were excluded because of possible measuring errors (N = 360).

### 4. Co-variables

The percentage of households in each PSU with any children (individuals less than 10 years old), adolescents (individuals between 10 and 20 years old), and elderly members (individuals age 65 years or older) was calculated. We also calculated the percentage of adults with underweight or excess weight (overweight and obesity) in each PSU.

Per capita household income was calculated as the total monthly household income divided by the number of individuals in the household. It included both monetary and non-monetary sources of income, including donations, gifts, self-production. We also calculated the mean per capita household income in each PSU.

Years of schooling of the head of the family was calculated using school attendance (i.e., if he/she had ever gone to school) and the last grade attended. Then, we calculated the mean years of schooling for the head of the families in each PSU. We also evaluated the PSUs' mean age of the head of the family.

### Analyses

Characteristics of PSUs by terciles of DDS were tested by trend analyses, as was the per capita amount of unhealthy diet markers (sugar, sweets, sugar-sweetened beverages, and crackers, cookies and cakes) acquired in the PSUs.

Multivariate linear regression models were used to calculate the association between the percentage of adults with underweight or excess weight in each PSU (dependent variables) and the PSU's terciles of DDS. Analyses were further adjusted for the number of individuals in the PSU, the percentage of households in each PSU with children or elderly member, the PSUs' mean age of the head of the family and PSU's per capita acquisition of sugar, sweets, sugar-sweetened beverages, and crackers, cookies, and cakes (Model 1) and further adjusted for mean per capita household income in each PSU (Model 2).

All percentages were weighted and performed, taking into account the sample design effect, using the survey procedure of the SAS system, version 9.1.

## Results

The PSU's DDS ranged from 5 to 23 healthy food groups; 5 to 19 in the first DDS tercile; 20 to 21 in the second tercile and 22 to 23 in the third tercile. The food groups most frequently purchased by the PSUs were bread, eggs and milk, whereas coconuts and nuts were purchased with the lowest frequencies. Potatoes were purchased more frequently than other roots and tubers, including manioc, cassava, and yam (Table 1).

The number of households in the PSUs varied from 4 to 21 (mean = 10.0), and the average number of individuals in each PSU was 35.8. These numbers increased with terciles of DDS. The per capita household income and the age and years of schooling of the head of the family increased with terciles of DDS. Frequency of households with at least one child decreased with DDS terciles, whereas the frequencies of households with at least one adolescent and with at least one elderly member did not differ by terciles of DDS. Regarding the anthropometric characteristics, PSU's in the higher DDS presented greater frequency of adults with obesity, while the frequency of underweight adults decreased according to PSU's DDS (Table 2 and 3).

**Table 2:** Characteristics of the Primary Sample Units - mean, frequency, 95% confidence interval (95% CI) and minimum and maximum

Demographics and socioeconomics characteristics	Mean or frequency (95% CI)	Minimum- maximum
Number of households (mean)	10.0 (9.9-10.1)	4.0-21.0
Number of individuals (mean)	35.8 (35.1-36.5)	7.0-93.0
Per capita household income (mean in U$)	201.3 (188.1-214.4)	17.1-1743.7
Age of the head of the family (mean)	46.4 (45.9-46.8)	27.6-68.1
Years of schooling of the head of the family (mean)	6.6 (6.4-6.8)	0.2-16.3
Frequency of household (%) with at least 1 child	37.9 (36.7-39.1)	0.0-100
Frequency of household (%) with at least 1 adolescent	43.5 (42.4-44.6)	0.0-100
Frequency of household (%) with at least 1 elderly member	17.9 (16.9-18.8)	0.0-90.0

Anthropometric characteristics		

Frequency (%) of adults with underweight	3.5 (3.2-3.7)	0.0-40.0
Frequency (%) of adults with overweight	26.5 (25.8-27.2)	0.0-75.0
Frequency (%) of adults with obesity	10.1 (9.6-10.6)	0.0-66.7

Brazil - urban area, 2002-2003.

**Table 3:** Characteristics of the Primary Sample Units by terciles of the dietary diversity score - mean, frequency and 95% confidence interval (95% CI)

	1^st ^tercile N = 1,162	2^nd ^tercile N = 1,214	3^rd ^tercile N = 1,017	p-value of trend
Demographics and socioeconomics characteristics				

Number of households (mean)	9.1 (8.8-9.3)	10.1 (9.8-10.4)	10.7 (10.5-10.9)	<0.0001
Number of individuals (mean)	33.0 (31.8-34.3)	36.1 (34.8-37.3)	37.8 (36.7-38.8)	<0.0001
Per capita household income (mean in U$)	141.7 (125.3-158.1)	201.4 (179.4-223.3)	251.7 (226.1-277.3)	<0.0001
Age of the head of the family (mean)	45.3 (44.6-46.0)	46.7 (45.9-47.5)	46.9 (46.2-47.6)	0.003
Years of schooling of the head of the family (mean)	5.7 (5.4-6.0)	6.5 (6.2-6.8)	7.4 (7.1-7.8)	<0.0001
Frequency of household (%) with at least 1 child	41.5 (39.5-43.6)	38.0 (36.0-40.0)	34.7 (32.7-36.7)	<0.0001
Frequency of household (%) with at least 1 adolescent	43.9 (42.0-45.8)	43.5 (41.6-45.4)	43.2 (41.4-45.0)	0.62
Frequency of household (%) with at least 1 elderly member	16.3 (14.9-17.6)	18.7 (16.9-20.5)	18.3 (16.8-79.9)	0.06

Anthropometric characteristics				

Frequency (%) of adults with underweight	4.1 (3.5-4.6)	3.3 (2.9-3.7)	3.2 (2.8-3.6)	0.01
Frequency (%) of adults with overweight	25.1 (23.7-26.4)	27.4 (26.2-28.6)	26.7 (25.5-27.9)	0.10
Frequency (%) of adults with obesity	9.0 (7.8-10.1)	10.5 (9.7-11.3)	10.6 (9.8-11.4)	0.05

Brazil - urban area, 2002-2003.

Greater acquisition of unhealthy foods (sugar, sweets, sugar sweetened beverages, and crackers, cookies and cakes) was associated with higher DDS (Table 4).

**Table 4:** Per capita acquisition in grams of sugar, sweets, sugar sweetened beverages, and crackers, cookies and cakes by terciles of the dietary diversity score - mean and 95% confidence interval (95%CI)

Acquisition (g/per capita/day)	Total N = 3,393	1^st ^tercile N = 1,162	2^nd ^tercile N = 1,214	3^rd ^tercile N = 1,017	p-value of trend
Sugar	55.1 (52.8-57.5)	50.5 (46.5-54.5)	56.1 (51.9-60.2)	57.7 (53.8-61.6)	0.02
Sweets	12.1 (11.3-13.0)	8.4 (7.0-9.7)	12.1 (10.8-13.3)	14.9 (13.5-16.4)	<0.0001
Sugar sweetened beverages	87.1 (82.9-91.4)	66.8 (60.7-72.9)	89.8 (82.3-97.2)	100.4 (93.0-107.8)	<0.0001
Crackers, cookies and cakes	16.9 (16.2-17.6)	13.1 (11.9-14.4)	17.2 (16.1-18.3)	19.6 (18.6-20.7)	<0.0001

Brazil - urban area, 2002-2003.

A higher PSU diversity was positively related to excess weight and negatively related to underweight. After controlling for the acquisition of unhealthy food items and mean per capita household income, the strength of associations was loosen, but they remained statistically significant (Table 5).

**Table 5:** Linear regression coefficient (β) of percentage of adults with underweight or excess weight in the Primary Sample Units regressed on terciles of Dietary diversity score (DDS)

	Without adjustments		Model 1*		Model 2†	
	**β**	**p-value**	**β**	**p-value**	**β**	**p-value**
						
Frequency (%) of adult with underweight in the PSU	-0.45	0.01	-0.45	0.01	-0.38	0.04
Frequency (%) of adults with excess weight in the PSU	1.52	0.003	0.89	0.08	0.98	0.05

Brazil - urban area, 2002-2003.

*Model 1: Adjusted for the number of individuals in the PSU, the percentage of households in each PSU with children or elderly member, PSUs' mean age of the head of the family and PSU's per capita acquisition of sugar, sweets, sugar-sweetened beverages, and crackers, cookies and cakes

†Model 2: Model 1 + mean per capita household income in each PSU

## Discussion

Although DDS was based on the acquisition of healthy food groups, as recommended by the Brazilian Food Guidelines, the frequency of adults with obesity was higher in the highest DDS tercile. Thus, our results also indicate that a high availability of healthy food groups does not mean low availability of unhealthy food groups. A possible explanation for the high acquisition of both healthy and unhealthy foods may be due to the many brands of foods introduced into the market every year. In general, unhealthy products provide high palatability, which has an important influence on energy intake [20,21] and some of these unhealthy foods are shaped, labelled and marketed as natural foods. Also, the great commercial appeal promoting *new foods *is habit-forming [22].

In Brazil, the household availability of soft drinks, crackers and cookies increased more than 400% between 1974-1975 and 2002-2003 [3]. Thus, our hypothesis that a high household availability of healthy dietary markers would decrease the availability of sugar, sweets, sugar-sweetened beverages and cracker and cookies was not confirmed. It seems that families do not replace the purchase of unhealthy foods with healthy foods, or vice-versa. Consistent with our finds, a community-based study of 90 American households showed that higher income households spent more dollars per person on both healthful and less healthful foods compared with lower income households [23].

Public policies related to nutrition have historically recommended a varied or diverse diet based on the fact that a single food item does not contain all the nutrients. Varied diet reduces the risk of developing nutritional deficiencies, as shown in other studies [12,13] and in this analysis, but dietary variety is also associated with higher energy intake, overweight and obesity [10,11,15]. The concept of variety and diversity may have lead people to add unhealthy foods in the diet, thus this behaviour increases intake of energy, fats, sweets and refined grain. On the other hand, a monotony diet is associated with lower energy intake [5,15]. In this line, the new United States dietary guidelines, for the first time, advocate consumption of fewer calories of a healthy diverse diet [24].

As expected, DDS was directly related to socioeconomic variables. Income and years of schooling of the head of the family increased with the PSU's DDS tercile. It has already been shown that there is a strong relationship between dietary diversity and household socioeconomic characteristics, and that increasing food expenditure results in a more diverse diet [8,25].

Supporting our finds related to the association between DDS and excess weight, Ponce and colleagues (2006) studied 325 Mexican men between the ages of 35 and 65 years and found a strong relationship between a diverse diet and the intake of total energy from fat and saturated fat. Also in this study, individuals with a more diverse diet presented higher intakes of cholesterol and were less in accordance with recommendations for preventing chronic diseases [26]. Among female university students, the association between DDS and energy intake was due to an increase in energy intake from fruits, vegetables and whole grains, indicating that increasing DDS is achieved by consuming more of these healthy food items. In these young females, those in the top quartile of DDS reported the lowest level of fast food intake [27]. However, two studies conducted in Tehranian adults by Azadbakht and colleagues (2005, 2006) showed an inverse association between DDS and hypertension, hypercholesterolemia, high LDL-C and diabetes [28,29].

Other studies also showed different results. Vandevijvere et al. (2010) did not find a relationship between DDS and BMI among either men or women [19]. Torheim and colleagues (2004) did not find an association between DDS and the nutritional status of adults [30], while Savy et al. (2005) found a significant association between dietary diversity scores and women's nutritional status, measured as BMI or body fat percentage [9]. In contrast, Azadbakht and Esmaillzadeh (2010) found an inverse association between DDS with obesity and abdominal adiposity in young females [27].

A possible explanation for controversies between the findings of the present study and the literature could be related with different definitions of DDS. DDS can be built in different ways depending on nutritional aspects and local food culture. Most dietary diversity measures consist of summing up the number of foods or food groups consumed over a seven-day period [6]. In our study we used information regarding all family food purchased for home consumption during a one-week period, a measure that reflects the household access to a variety of food groups [6,8]. The relative validity of this measure was indicated by Hoddinott and Yohannes (2002) who found a positive association between dietary diversity with per capita expenditure and with total per capita energy availability [8]. However, additional research is necessary to determine which food groups should be included in the DDS [6].

In developing countries with a poor socioeconomic situation and high prevalence of underweight, overall diversity is protective [9,12,13]. Similarly, we found a negative association between diversity and frequency of households with any underweight adult.

Studies in countries with different socioeconomic structures, such as Africa [11] and Belgium [19], included separate analyses with and without taking into account the energy-dense food groups. In both studies, it did not change the interpretation of the results; dietary diversity is a good indicator of nutritional adequacy. In our study, we separately evaluated the availability of sugar, sweets, sugar-sweetened beverages, and crackers, cookies and cakes because our main purpose was to investigate whether a decrease in the availability of these low-nutrition foods would be seen when purchases indicated a high DDS.

To our knowledge, this is the first study using HBS to access the diversity of diets. Purchases could be more informative then individual intake because they reflect the range of foods that are available to the household, but the majority of studies on diversity use conventional dietary assessment methods. Underreporting in individual methods of diet evaluation is also high, mainly among overweight and obese individuals [31], a factor overcome by analyzing purchases. Also, the availability of foods across seasons was not a major issue in our study because the time frame was a one-year period.

The main limitation of HBS data is that the diversity score at the household level does not reflect all food consumption because eating out of the home is not included. However, a previous analysis of this database indicated that the majority of the items consumed away from home were not healthy items included in our DDS [32]. Food distribution within families is also a limitation in our analysis [33].

In line with our findings a review of diet quality score in many developed countries also concluded that healthy dietary scores are not good predictor of health outcomes [34].

## Conclusions

Dietary diversity may not reflect an option for a healthy diet because a high diversity of healthy items is correlated with the availability of unhealthy food items. On the other hand, the association of diversity with a lower prevalence of underweight confirms diversity as a good marker among populations with low access to food. Because our data indicate that there is no replacement of unhealthy food groups with healthy food groups, a healthy diet message for obesity prevention should be necessarily combined with a message that focuses on eating less.

## Competing interests

The authors declare that they have no competing interests.

## Authors' contributions

INB contributed to analysing and interpreting the data, and drafting the manuscript. RS conducted data analysis and interpretations and all authors read and approved the final manuscript.
